# Appetite and Protein Intake Strata of Older Adults in the European Union: Socio-Demographic and Health Characteristics, Diet-Related and Physical Activity Behaviours

**DOI:** 10.3390/nu11040777

**Published:** 2019-04-04

**Authors:** Yung Hung, Hanneke A. H. Wijnhoven, Marjolein Visser, Wim Verbeke

**Affiliations:** 1 Department of Agricultural Economics, Ghent University, Coupure links 653, 9000 Ghent, Belgium; wim.verbeke@ugent.be; 2 Department of Health Sciences, Faculty of Science, Vrije Universiteit Amsterdam, Amsterdam Public Health research institute, De Boelelaan 1085, 1081 HV Amsterdam, The Netherlands; hanneke.wijnhoven@vu.nl (H.A.H.W.); m.visser@vu.nl (M.V.)

**Keywords:** aged, consumer, eating behaviour, elderly, food, geriatric health, physical activity, protein-energy malnutrition (PEM), segmentation

## Abstract

Considerable efforts have been directed towards stimulating healthy ageing regarding protein intake and malnutrition, yet large-scale consumer studies are scarce and fragmented. This study aims to profile older adults in the European Union (EU) according to appetite (poor/good) and protein intake (lower/higher) strata, and to identify dietary and physical activity behaviours. A survey with older (aged 65 years or above) adults (*n* = 1825) in five EU countries (Netherlands, United Kingdom, Finland, Spain and Poland) was conducted in June 2017. Four appetite and protein intake strata were identified based on simplified nutritional appetite questionnaire (SNAQ) scores (≤14 versus >14) and the probability of a protein intake below 1.0 g/kg adjusted BW/day (≥0.3 versus <0.3) based on the 14-item Pro55+ screener: “appi”—Poor appetite and lower level of protein intake (12.2%); “APpi”—Good appetite but lower level of protein intake (25.5%); “apPI”—Poor appetite but higher level of protein intake (14.8%); and “APPI”—Good appetite and higher level of protein intake (47.5%). The stratum of older adults with a poor appetite and lower level of protein intake (12.2%) is characterized by a larger share of people aged 70 years or above, living in the UK or Finland, having an education below tertiary level, who reported some or severe financial difficulties, having less knowledge about dietary protein and being fussier about food. This stratum also tends to have a higher risk of malnutrition in general, oral-health related problems, experience more difficulties in mobility and meal preparation, lower confidence in their ability to engage in physical activities in difficult situations, and a lower readiness to follow dietary advice. Two multivariate linear regression models were used to identify the behavioural determinants that might explain the probability of lower protein intake, stratified by appetite status. This study provides an overview and highlights the similarities and differences in the strata profiles. Recommendations for optimal dietary and physical activity strategies to prevent protein malnutrition were derived, discussed and tailored according to older adults’ profiles.

## 1. Introduction

Population ageing has been a major social and economic challenge facing the European Union (EU). Owing to the affluent healthcare systems, life expectancy continues to grow in many countries at a different pace. In the EU, 19.4% of the population were aged 65 years or older in 2017 [1]. This demographic development has received increasing attention from policy officials and commercial sectors with an interest to address the health needs of older adults [2].

An important concern in older age is related to protein intake and malnutrition, as it is associated with negative consequences for health, physical functioning and quality of life that impose a high burden on health care use and costs [3,4,5,6,7,8]. Considering the complex nature of malnutrition development and the irreversible consequences, a holistic approach towards prevention is essential [9]. At the moment, there is no simple solution that effectively halts protein malnutrition, as protein or amino acid supplementation alone does not contribute sufficiently to increasing muscle mass or strength in older adults [10]. Increased boredom and decreased liking of oral supplements due to repeat consumption constitute an obstacle to compliance within this type of intervention or treatment [11]. Furthermore, older individuals tend to be more sceptical towards foods enriched with protein compared to the younger group [12]. A combination of anabolic stimuli such as adequate protein intake and physical activity is commonly regarded as the most promising strategy to prevent protein malnutrition and its consequences [13], albeit a reduced responsiveness to these anabolic stimuli in older age [14]. Age-related changes in protein metabolism and weakened anabolic response to ingested protein increase the need for protein in healthy older adults [15]. Therefore, the European Society for Clinical Nutrition and Metabolism (ESPEN) recommends a daily protein intake of 1.0–1.2 g protein per kg body weight in healthy individuals over 65 years [16]. Recent studies concluded that there is a high prevalence of older adults at risk of having protein intake below 0.8 g protein per kg body weight [17,18,19]. Thus, it remains a target of interest to identify and assess the determinants of a lower protein intake, so as to optimize dietary and physical activity strategies that may help prevent protein malnutrition in older adults.

The process of ageing brings about physiological, psychological and social changes that affect food choice and consumption [20,21,22]. Physiological changes such as weakened sense of taste, declined olfactory function, and/or difficult chewing and swallowing may have a negative impact on appetite and food intake in older adults [23], and hence, influence protein intake from food [2,21]. Psychological changes, for instance, reduced inhibitory capacity, increased aversion to innovativeness, and/or increased vulnerability to confirmatory search processes make older adults more prone to rely on habitual food choice and consumption pattern [24,25,26]. Social factors such as living conditions and family situation have an additional influence on food and nutrition intake [27]. For example, living alone has been shown to be negatively associated with protein intake or overall diet quality in older adults [20,28]. Apart from food consumption and diet, these age-related changes also pose barriers for another important anabolic stimulus—physical activity [29], making the prevention of protein malnutrition a challenge. Considerable scientific efforts have been directed towards the key to healthy ageing regarding protein intake and malnutrition, yet the complicated picture remains unsolved.

Large-scale studies related to the associations of physiological, psychological and social factors with protein intake are scarce and fragmented, particularly regarding how factors interact in relationship with protein intake [22,30,31,32]. Therefore, the present study aims to explain the probability of lower protein intake in older adults in the EU, as food consumers, using a holistic approach. There is a larger proportion of institutionalized older adults who are malnourished compared to community-dwelling subjects. Nevertheless, the majority (90–95%) of older European citizens lives at home [33], and thus, there is a much larger absolute number of malnourished community-dwelling older adults compared to those who are institutionalized [34]. Hence, our study focuses on community-dwelling older adults. Community-dwelling older adults also have more personal control over their food purchase and consumption decisions, and thus are a large group of consumers who can deliberately opt for and benefit from food-related innovations stimulating healthy ageing.

Van der Meij et al., suggested that protein malnutrition should be studied with appetite, as appetite is strongly linked to the actual protein intake [35]. Older adults with poor appetite tend to have a lower protein intake, which increases their risk of protein—energy malnutrition [36] and sarcopenia [37]. Since older adults with different appetite and protein intake profiles have different nutritional needs and dietary intake patterns [35], they should be taken into account as different subgroups in order to cater for their specific needs.

Hence, the objectives of this study were threefold. The first objective was to identify the appetite and protein intake strata, which form the basis for profiling the older adults with regard to their demographic, socioeconomic, health, knowledge, attitudinal, diet-related and physical activity characteristics. As a result, the potential target group can be identified and profiled for new food product development and related public health and marketing efforts. Given the important role of habit in older age [24,25,26], the second objective was to examine how or with what kind of food products the target group’s protein intake status might be improved, by identifying the habitual dietary and physical activity behaviours that shape protein intake, while accounting for appetite. Finally, based on the above insights, the third objective was to formulate implications and recommendations for optimal dietary and physical activity strategies for the prevention of protein malnutrition that meet the specific health needs and match with the preferences and habits of the targeted senior consumers.

## 2. Materials and Methods

### 2.1. Study Design and Sampling

Cross-European data were collected in June 2017 through a cross-sectional quantitative online survey in five EU countries: (United Kingdom, Finland, the Netherlands, Spain and Poland; *n* = ±365 per country). In the EU, about half of the older population (aged 65–74 years) used the internet at least once a week in 2016 [38]; therefore, an online survey could reach a substantial amount of community-dwelling older adults. A total of 1825 participants were recruited by means of probabilistic sampling from the online access proprietary panel of a professional market research agency, which abides the ICC/ESOMAR International Code on Market and Social Research regarding ethics in social sciences research. Recruitment criteria were set for older adults (65 years or above) who live independently. Additional measures were in place to achieve a nationally representative sample in terms of gender and region in each of the study countries, following a standard procedure: (1) the panellists were selected based on the background information collected during the registration survey and ongoing profiling and screening surveys; (2) specified quotas were set for gender (50% female and 50% male) and regions proportional to the population distribution; (3) the panellist were invited in batches at designated times with close monitoring during the fieldwork. All procedures for contact and questionnaire administration were electronic via the same market research agency. Ethics approval for the study was granted by the Belgian Ethics Committee of Ghent University Hospital in March 2017 (Reference No. B670201422567). All collected data were coded in a non-identifiable format and processed anonymously.

### 2.2. Questionnaire Content and Pre-Testing

The questionnaire was developed in English and translated into the respective national languages, beside English for the UK; Dutch for the Netherlands, Finnish for Finland, Spanish for Spain and Polish for Poland by a contracted professional translation office. The translated versions of the questionnaire were proofread by native speakers of the respective languages who were affiliated with the research consortium. The questionnaires were pretested by the market research agency and the involved researchers in a sample of about 30 consumer panel members for clarity of content, language and wording, overall understanding and length of the survey. Based on this pre-test and feedback the questionnaire was refined and finalised.

The survey began with a short description of the EC-funded project—PROMISS (“PRevention Of Malnutrition In Senior Subjects in the EU”) and the informed consent; this was followed by a screening for sample selection based on gender, age, region and current living condition. The core questions consisted of eight sections: appetite, dietary habits, physical activity, food-related and physical activity attitudes, knowledge and perception of protein and food in the diet, socio-demographics and personal information. Order bias was avoided by rotating items within a question.

Appetite was measured using the simplified nutritional appetite questionnaire (SNAQ) by Wilson et al. [39]. The SNAQ had been validated by Young et al. [40] and Hanisah, Shahar and Lee [41]. It consisted of four questions that participants had to indicate the answer that could best apply to their current situation e.g., “My appetite is…”, with responses on a five-point-scale ranging from “very poor” (=1) to “very good” (=5). SNAQ score was calculated based on the numeric scale (i.e., from 1 to 5) assigned to the choices of each question. Poor appetite was defined as having a total SNAQ score below or equal to 14, as an individual with SNAQ ≤14 has a significant risk of weight loss of more than 5% within 6 months with a sensitivity of 81.5% and a specificity of 76.4% [42].

Protein intake was estimated using the Protein Screener 55+ (Pro55+) (Vrije Universiteit Amsterdam, Amsterdam, the Netherlands), which is a 14-item questionnaire based on the HELIUS (HEalthy LIfe in an Urban Setting) food frequency questionnaire [43,44]. The questions include the consumption frequency and portion size in relation to nine food items, e.g., “In the last 4 weeks, how many slices/pieces of bread did you eat on an average day?” where participants could indicate the amount from none/less than 1 to more than 12. A lower level of protein intake was defined as having a probability higher than 0.3 that the protein intake was lower than 1.0 g per kilogram of adjusted body weight per day (g/kg adjusted BW/day) based on recalibrated models [44].

The consumption pattern of 10 food items (cereals like cornflakes or muesli, dairy or plant-based milk or yogurt, soup, warm meal, cold meal, dessert, biscuits or cookies, fruits, nuts or seeds) was recorded by means of check-all-that-apply (CATA) questions for seven eating occasions (“breakfast”, “mid-morning snack”, “lunch”, “mid-afternoon snack”, “dinner”, “evening snack”, “nocturnal eating”) or “I do not consume this food”. Appendix A Table A1 presents the frequency table for the consumption pattern of the 10 food items.

Physical activity (PA) was measured using the short version of the International Physical Activity Questionnaire (IPAQ) [45], in which the questions were adapted for older adults and validated by Hurtig-Wennlöf, Hagströmer and Olsson [46]. For example, “Think about the time you spent sitting during the last 7 days, including time spent at home, while doing housework and during leisure time. This may include time spent sitting at a desk, visiting friends, reading, or lying down or sitting to watch television. During the last 7 days, how much time did you spend sitting during a day?” and participants were asked to indicate the hours and minutes. The IPAQ scores were calculated following the guidelines given by the IPAQ Research Committee [47]. Apart from the IPAQ, participants also had to indicate the moment of the day for the physical activity with CATA questions (“before breakfast”, “between breakfast or and lunch”, “between lunch and dinner” and “after dinner”), whether they were willing to change their daily physical activity pattern and duration of sleeping. Short sleepers were defined as individuals who sleep less than or equal to 6 h per night and long sleepers were people who reported to sleep more than 8 hours per night [48].

The definition of physical activity according to the National Institutes of Health was shown to the participants prior to the question of self-efficacy to engage in PA, i.e., “physical activity is any body movement that works your muscles and requires more energy than resting (lying down or sitting). Walking, running, swimming, yoga, all types of sports, and housework like cleaning and gardening are a few examples of physical activity”. Self-efficacy is defined as one’s confidence in their ability to engage in physical activities under various situations, and it appeared to be a stronger predictor to actual behaviour than attitudes towards physical activity [49]. Self-efficacy was measured using 15 items modified based on Bandura [50] with the validation and item combination suggested by Everett, Salamonson and Davidson [51]. For example, participants could rate how confident they were on a five-point-scale from “not at all confident” (=1) to “extremely confident” (=5), or “not applicable” (=missing value) to a statement such as “I am confident in my ability to engage in physical activities when I am feeling tired”.

Participants were asked whether they know what dietary protein is. If “no” was indicated, the definition of dietary protein was given. If “yes” was indicated, they were directed to an objective knowledge test. Knowledge and perception of protein was evaluated by means of objective knowledge items, wherein correct and incorrect statements were presented (e.g., “You need protein in the diet for energy”) and participants had to answer one of the three choices “True”, “False” or “I do not know” (Appendix A Table A2). The option of “I do not know” was included so as to reduce the level of bias induced by guessing.

Food fussiness was measured with six items adapted from Wardle et al. [52] which were also previously tested in a sample with older adults [53]. For example, “I enjoy tasting new foods” and the responses were on a five-point-Likert scale from “strongly disagree” (=1) to “strongly agree” (=5). Participants were also asked to indicate what they think about the amount of protein in their daily diet (i.e., on a five-point-scale ranging from “Too much” (=1) to “Too little” (=5) or “I do not know” (=missing value)), and whether they intend to change the amount (i.e., on a three-point-scale from “Yes, increase the amount” (=1) to “No, remain the same” (=3) or “I do not know” (=missing value)). In addition, participants were asked if they would increase the amount of protein in their diet if they were told by a health professional, food industry, family or friends (i.e., “Yes”, “No” and “I do not know”).

A series of 17 questions were used to assess the personal characteristics of participants, which included socio-demographics such as education level, being the main household (HH) grocery shopper, HH income, food expenses, lifestyle such as smoking and alcohol use, health characteristics such as risk of malnutrition, mobility, ability in preparing own foods, presence of various health problems, etc. The risk of malnutrition was measured using the Malnutrition Universal Screening Tool (MUST) [54] with five questions and body mass index (BMI), which was evaluated and validated by Poulia et al. [55] and Leistra et al. [56] and found to have high sensitivity. For example, “Has your intake of food been poor for the last 5 days or likely to be poor for the next 5 days?” and participants were asked to indicate “Yes” or “No”.

### 2.3. Statistical Analysis

Statistical analyses were carried out with SPSS Statistics 25.0 (IBM SPSS, Armonk, NY, USA). Data processing and analysis included descriptive analysis (frequency distributions), bivariate ANOVA or chi-square tests (comparison of consumers’ characteristics across the appetite and protein intake strata) and multivariate analysis (calculation of the probability of lower protein intake and identification of its behavioural determinants).

#### 2.3.1. Appetite and Protein Intake Strata

The appetite and protein intake strata were defined based on the SNAQ scores and probability of lower protein intake. The calculation of the probability of having a lower protein intake was performed based on multivariate logistic regression analysis, for which the protocol and algorithm have been reported in Wijnhoven et al. [44]. There were 156 older adults who did not report their body weight (8.5% of the sample), while this information is required for the calculation of the probability of lower protein intake. Body weight was first adjusted for overweight and underweight individuals based on age and height (BMI), in which adjusted bodyweight was the nearest required for the individuals to have a BMI in the healthy range, i.e., 18.5–24.9 for adults aged ≤70 years and 22.0–27.0 for adults aged ≥71 years [57]. Since adjusted body weight was used in the calculation of the probability of lower protein intake, the variation in body weight has relatively low leverage over the calculated probability. Therefore, instead of excluding the cases with missing body weight, the missing values were imputed with a fully conditional specification. Multiple imputation was performed using age, gender, country, education level, smoker status, height, physical activity level (IPAQ), self-reported presence of overweight and self-reported presence of underweight as predictor variables. The same analyses have been performed with missing data on the bodyweight excluded. The main conclusions remained unchanged, as the proportion of the strata and variables selected in the linear regression models did not differ substantially.

Poor appetite was defined as having a total SNAQ score below or equal to 14; low level of protein intake was defined as having a probability of protein intake lower than 1.0 g/kg adjusted BW/day equal to or higher than 0.3. The strata were profiled and compared in terms of older adults’ socioeconomic and demographic background, health characteristics, presence of health problems, knowledge and attitude related to protein, food and diet and attitude towards physical activity. Effect sizes, Cramer’s phi (ϕc) or partial eta-squared (η_p_^2^) were included in the analyses to support the interpretation of the *p*-value for which very low values can be obtained as a result of large samples sizes as in this study. Effect sizes indicate the proportion of variance in the variable (e.g., strata) explained by another variable (e.g., socio-demographics) and as such, indicate the strength of a relationship between variables and the significance of differences between strata [58]. Cramer’s phi was computed for chi-square tests and considered small when in the range 0.1–0.3, medium from 0.3 and large from 0.5 [59]. Partial eta-squared was computed for Kruskal–Wallis one-way analyses of variance and considered small from 0.01, medium from 0.06 and large when equal to or greater than 0.13 [60]. For chi-square association tests, the test was not considered reliable if more than 20% of the cells had expected counts of less than five, as a large amount of sparse cells does not allow a valid comparison [61].

#### 2.3.2. Behavioural Determinants of Protein Intake

Two multivariate linear regression models were used to identify the behavioural determinants that might explain the probability of lower protein intake (continuous scale), while stratifying by appetite. Model 1 considered older adults who have reported poor appetite (SNAQ score ≤ 14; *n* = 493), while Model 2 included older adults with good appetite (SNAQ score > 14; *n* = 1332).

The explanatory variables included a series of possible behavioural determinants in terms of dietary habit (e.g., food expenses, consumption frequency and moment of certain food groups or products, diet status, etc.) and physical activities (e.g., physical activity levels and pattern). Categorical variables were coded as dummy variables for comparison. Expenses on food per week (for consumption at home and out-of-home) had four levels (i.e., less than 60 EUR, between 60 and 119 EUR, 120 EUR or above, prefer not to say or do not know); the level “between 60 and 119 EUR” was set as the reference category while the others were coded as dummy variables. Physical activity level based on IPAQ had three levels (i.e., high, medium and low). For example, a high level of physical activity denotes at least five days of any combination of walking, activities of moderate or vigorous intensity per week; moderate level denotes at least three days of vigorous activity of at least 20 min per day; low level denotes no or very low (insufficient to meet moderate or high level of) physical activity reported. The scoring protocol and categorical levels are reported in the guideline published by the IPAQ Research Committee [47]. High physical activity level was set as the reference category while the other levels were coded as dummy variables.

All major assumptions have been tested for the multivariate linear regression model. There was no issue of multicollinearity. Collinearity diagnostics included: no values of variance inflation factor (VIF) larger than 10; no average VIF-values substantially larger than 1 (the largest VIF value: 1.234 in Model 1 and 1.248 in Model 2); no tolerance value below 0.2 (the smallest tolerance value: 0.810 in Model 1 and 0.801 in Model 2); no correlation coefficient between two explanatory variables in the model larger than 0.50. The plot of standardized residuals against standardized predicted values for both models showed a slight tendency of funnelling out but no curve formation, signalling there could be heteroscedasticity in the data. Yet, the assumption of linearity has been fulfilled. The distribution of errors was close to normal. The results of the assumption tests have shown that multivariate linear regression is a suitable statistical analytical method for this study and its data. In order to account for possible issues of assumption violation, a bootstrap method was applied to provide more robust statistics.

There were eight potentially influential cases identified in Model 1 and 69 potentially influential cases in Model 2 based on several parameters: covariance ratio (CVR) (cases with CVR >1 + (3(k + 1)/*n*) or <1 − (3(k + 1)/*n*); Model 1: CVR >1.073 = 2 cases and <0.927 = 6 cases; Model 2: CVR >1.029 = 24 cases and <0.970 = 45 cases); standardized residuals (2.64% (Model 1) and 5.03% (Model 2) of cases have absolute values above 2, and 0.20% (Model 1) and 1.28% (Model 2) have absolute values above 2.5, any case with the value above about 3, could be an outlier: 0 cases (Model 1) and 1 case (Model 2)); all cases have Cook’s distance value lower than 1; the average leverage (cases >3 × ((k + 1)/*n*) = 0.073:0 cases (Model 1) and 0.029:0 cases (Model 2)); no absolute values of DF-Beta was greater than 1. Linear regression models were run with identical outcome and explanatory variables, both with and without the influential cases removed. The resulting models were similar in terms of retained variables; however, the adjusted *R*^2^ showed a reasonable improvement in the model fit after the removal of outliner (one case in Model 2) and influential cases (eight cases in Model 1 and 69 cases including the outliner in Model 2). The adjusted *R*^2^ increased from 17.7% to 20.6% in Model 1 and from 15.7% to 21.9% in Model 2. Therefore, all potentially influential cases were removed in the final models (Model 1: *n* = 485; Model 2: *n* = 1263).

## 3. Results

### 3.1. Appetite and Protein Intake Strata

**Figure 1:**
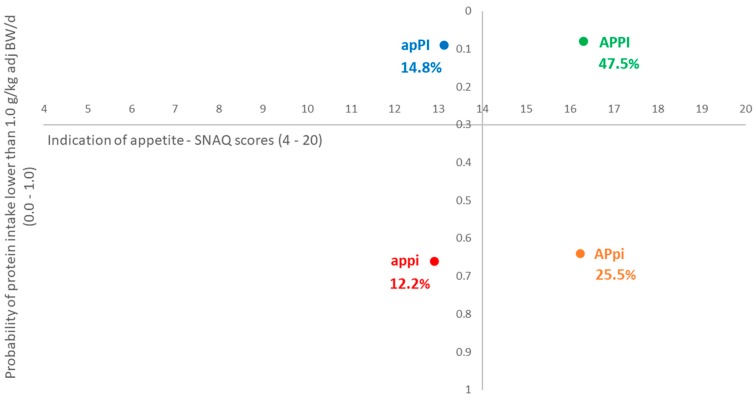
Positioning of the four appetite and protein intake strata based on their median values for the appetite scores (based on simplified nutritional appetite questionnaire) and probability of protein intake lower than 1.0 g/kg adjusted BW/day (*n* = 1825). appi: poor appetite and lower level of protein intake; APpi: good appetite but lower level of protein intake; apPI: poor appetite but higher level of protein intake; and APPI: good appetite and higher level of protein intake.

Based on the SNAQ scores and the predicted probability of lower protein intake, four appetite and protein intake strata were identified. The size and scores of the appetite and protein intake strata are shown in Table 1. Stratum 1 “appi” included 12.2% of the sampled older adults; they reported poor appetite and a lower level of protein intake. Stratum 2 “APpi” accounted for 25.5% of the older adults; they reported a good appetite but a lower level of protein intake. Stratum 3 “apPI” (14.8% of the sample) refers to the older adults who reported poor appetite, though a higher protein intake. Stratum 4 “APPI” represents the ideal situation for appetite (good) and protein intake (higher), which accounts for almost half (47.5%) of the total sample. Figure 1 illustrates the positions of the four strata based on their median values for the SNAQ scores and probability of protein intake lower than 1.0 g/kg adjusted BW/day. An overview and highlights of the similarities and differences in their profiles are provided in the following sections.

#### 3.1.1. Demographic and Socioeconomic Profile

The four strata did not differ significantly in terms of being the main household grocery shopper or living alone (Table 2). Meanwhile, appi was overrepresented by older adults who were 70 years or above, living in the United Kingdom or Finland, having an education below tertiary level, and who reported some or severe financial difficulties. APpi had a larger proportion of older adults who were male, from the United Kingdom or the Netherlands, perceiving their financial situation as quite well or very well, yet many having a net monthly income below 1000 EUR. The stratum apPI had more older adults who were from Spain or Finland, and who had a net monthly income between 1000 EUR and 2000 EUR. APPI was accounted for by a larger proportion of older adults who were female, from Poland or Spain, having a tertiary education level or above, and who always made their own food decisions.

#### 3.1.2. Health Characteristics and Problems

In terms of health characteristics, the two strata with poor appetite (appi and apPI) contained more older adults who had a high risk level of malnutrition in general, and were able to walk or move their own wheelchair for more than 5 min but with difficulties (Table 3). APPI was overrepresented by older adults who were able to walk or move their own wheelchair for more than 5 min and to prepare their own meal without difficulties. Regarding the presence of health problems, the patterns were not fully consistent. The two strata with poor appetite (appi and apPI) were also overrepresented by older adults who had oral health-related infirmities such as pain in the mouth, teeth or gum, dry mouth, difficulty chewing or swallowing, as well as diabetes or high blood sugar levels and other chronic disease or pain in general (Table 4).

#### 3.1.3. Knowledge and Attitude Related to Protein, Food and Diet

Concerning knowledge about dietary protein, the two strata with lower protein intake (appi and APpi) were overrepresented by older adults who reported not knowing what protein is and had a lower objective knowledge score about dietary protein, whereas the opposite findings were observed for APPI. Appendix A Table A2 presents the strata profiles based on the specific misconception about dietary protein. The majority of appi believed that one meal per day with a good protein source is sufficient, while APpi thought that 100 mL of whole milk has more protein than 100 g of cheese. The strata with poor appetite (appi and apPI) were accounted for by more older adults who were fussier about foods. A larger proportion of appi did perceive the amount of protein in their diet as too little, which is matching with their protein intake as estimated using the Pro55+ tool. Though apPI had a higher protein intake based on the Pro55+ tool, a larger proportion of them perceived the amount of protein in their diet as too little. APPI had more older adults who perceived the amount of protein in their diet as just about right and who had no intention to change the amount. All strata except APPI were overrepresented by older adults who did not know if they want to change their protein intake. The stratum appi was underrepresented by older adults who indicated that they would increase the amount of protein in their diet if health professionals told them to do so (Table 5).

#### 3.1.4. Attitude Towards Physical Activity

Regarding physical activity, the two strata with lower protein intake (appi and APpi) included more older adults who did not have a regular daily physical activity pattern, and also reported that being willing to change their daily physical activity is not applicable. The stratum appi had more older adults with a lower self-efficacy to engage in physical activity. The strata with higher protein intake (apPI and APPI) had larger proportions of older adults who were unwilling to change their daily physical activity pattern, while APPI tended to have a higher self-efficacy towards physical activity (Table 6).

### 3.2. Behavioural Determinants of Protein Intake

Habitual dietary and physical activity behaviours that are associated with protein intake were identified using two multivariate linear regression models. Model 1 included older adults who have reported a poor appetite (SNAQ score ≤14), and Model 2 included the older adults with a good appetite (SNAQ score >14). Both models explained 20.6% and 21.9% of the variance in the probability of having lower protein intake, respectively. With regard to behavioural determinants (Table 7), lower food expenditure and lower consumption frequency of specific food groups emerged as determinants for having a lower protein intake regardless the level of appetite. Consumption of certain foods at a certain moment of the day and physical activity level or pattern were associated with (a lower risk of having) a lower protein intake, which differed upon appetite status. The similarities and differences are described in the following sections.

#### 3.2.1. Diet-Related Habits

For older adults with poor appetite (Model 1), spending less than 60 EUR (compared to between 60 and 119 EUR) per week on food was associated with a lower protein intake. Furthermore, the probability of lower protein intake increased with the absence of milk or yogurt consumption, absence of nuts or seeds consumption, and consumption of dessert during dinner. On the other hand, the probability decreases with consumption of fruits during dinner and cold meal during evening snack moment.

Concerning older adults with good appetite (Model 2), the probability of lower protein intake increases with the absence of nuts or seeds consumption, milk or yogurt consumption, and consumption of fruit during evening snack moment. On the contrary, being a current smoker, consumption of warm meal during lunch, soup during mid-afternoon snack, milk or yogurt during mid-afternoon snack and cold meal during evening snack decreased the probability of having a lower protein intake.

#### 3.2.2. Physical Activity

For older adults with poor appetite (Model 1), a lower level of physical activity was associated with a higher probability of having a lower protein intake. The association of having a low physical activity level (compared to a high level) was two times stronger than having a moderate physical activity level. Level of physical activity does not matter in older adults with good appetite (Model 2), yet the probability of having a lower protein intake decreased when older adults performed vigorous physical activities between lunch and dinner.

## 4. Discussion

The present study sought to profile older adults from different European countries according to appetite and protein intake strata, and to identify the habitual dietary and physical activity behaviours that are associated with protein intake. Based on the empirical insights, recommendations for optimal dietary and physical activity strategies to prevent protein malnutrition were derived.

### 4.1. Demographic, Socioeconomic and Health Characteristics

The findings indicate that more efforts may be needed depending on the country, as the group of older adults at risk of lower protein intake is found in each of the study countries, but not to the same extent. The primary target markets (older adults at risk of having poor appetite and/or lower protein intake) for dietary strategies and new product development are situated in the United Kingdom (more than in, e.g., Spain). EU countries may differ largely with regard to their social and dietary culture and customs, which may have an influence on appetite and protein intake. Nevertheless, as a trade-off for reporting such an extensive amount of data in the current study, it was not feasible to further take into account the country differences. Yet, a better understanding of eventual country differences could contribute to the national policies and is of value for future research. In line with expectations and previous studies, being 70 years or above and having a lower education level emerge as risk factors for poor appetite in combination with a lower level of protein intake [17,28,35]. Previous findings related to gender and appetite and protein intake are inconsistent [35,62,63]; we found that males could be at a higher risk compared to females with regard to lower protein intake. Although Raatz et al., found that smokers have a tendency of having a lower protein intake [64], our results show that current smokers with a good appetite tended to have a higher protein intake, which was also reported in [62]. Contrary to expectations and previous studies [28,65], living alone was not associated with appetite status nor protein intake. Irz et al. [66] reported that insufficient resources such as income was not a driver for suboptimal dietary choices, which was partially confirmed by our findings that the strata did not differ with a consistent pattern in terms of household income level. Nonetheless, our study found that the highest risk stratum reported some or severe financial difficulties, while income was positively correlated with food expenditure and a lower food expenditure was an important risk factor for lower protein intake, regardless of appetite status. Therefore, economic constraints might be at stake.

In terms of health characteristics, poor appetite was closely linked to the risk of malnutrition and functional limitations such as walking or moving with a wheelchair for more than 5 min or being able to prepare their own meals, as previously reported [67,68]. Nyberg et al. mentioned in their review that eating difficulties such as problems with chewing or swallowing are important determinants of malnutrition [69]. Health profiles of the strata showed that oral health-related health problems seemed more prominent in older adults with poor appetite, regardless of their protein intake. Besides health characteristics, in line with expectations, older adults with poor appetite also reported to be fussier about food, i.e., to be less prone to accepting new (types of) food.

These findings suggest that dietary strategies to increase protein intake should pay attention to sensory properties so as to combat poor appetite, to familiarity to cope with food fussiness, to affordability to match with lower expenditure on foods, to accessibility to overcome the challenge of low mobility, as well as to convenience that fits older adults’ ability for meal preparation and ingestion.

### 4.2. Diet-Related Characteristics and Behaviours

Objective knowledge about dietary protein appears to be positively linked to protein intake. Older adults’ knowledge related to protein consumption was previously assessed by van der Zanden et al., using focus group discussions [70], in which the awareness of the importance of protein consumption was considerably high. Most older adults managed to list the food groups or products that are rich in protein (e.g., meat, eggs, legumes, etc.), but they seemed lacking knowledge in relation to the resulting physiological functions of protein [70]. Our results show that the most common misconceptions among the older adults were related to the amount of protein needed in their diet; the vast majority believe that one meal per day with a good protein source is sufficient. Moreover, almost half of the studied older adults hold misconceptions, such as that health experts recommend people of older age to consume less protein that the human body is able to store protein for a later use, and that it is thus not necessary to consume a steady amount of protein daily or that 100 mL of whole milk has more protein than 100 g of cheese. Apart from the actual knowledge about dietary protein, most older adults believed that the amount of protein in their current diet is just about right. Especially when they had a good appetite, they tended to think that they did not need to change the amount of protein intake. These findings shed light on possible key messages in communication strategies, in which not only the awareness about the importance of protein consumption should be increased, but the messages should also be specific in relation to the amount of protein needed and concrete actions on how recommended levels could be achieved. While interventions simply based on standalone information provision might not be the most effective [71], some evidence suggests that nutrition education by means of cooking demonstrations and distribution of food ingredients in the community can improve the nutrition status of older adults [72]. Considering the lower level of knowledge and intention to change the amount of protein intake in the higher risk strata, coupling this with interventions to increase knowledge appear to stand a fair chance of being effective. However, it remains a major challenge to reach the higher risk strata, as they have a lower readiness to follow dietary advices from health professionals, even though health professionals were rated as the most trusted information source.

With regard to habitual dietary behaviours, our findings touched upon two of the three aspects of chrononutrition [73]: timing and frequency of eating behaviours; regularity was not covered. Literature shows mixed results regarding the effects of timing, distribution and eating patterns on protein intake and physiological functions [74,75,76]. Earlier studies found that older adults with a lower protein intake tended to consume more of their total daily protein intake during the morning meals, which might be explained by greater satiety later in the day [17,77]. No association between the consumption of foods (any of the nine studied items) during breakfast or mid-morning snacks and protein intake was found in our results, yet it should be noted that the consumed amount was not taken into account.

Remarkable is that the association between fruit consumption (at different moments) and lower protein intake was opposite in the two models: fruit consumption during dinner decreased the risk of lower protein intake for older adults with poor appetite, whereas fruit consumption during lunch, or evening snack moment increased the risk for older adults with good appetite. It has been repeatedly reported that fruit consumption increases satiety, owing to the sugar and fibre content [78,79], which explains the result related to older adults with good appetite. This is contradictory to our finding related to older adults with poor appetite. Considering that the types of fruit consumed by the older adults were not known, two possible explanations may be linked to certain fruits’ high acidity and/or fructose content. Acidic foods stimulate increases in salivary flow [80]. Our results show that more than 20% of the older adults with poor appetite reported experience of dry mouth. As dry mouth is strongly associated with loss of appetite [81,82], consuming fruit with acidity may increase consumers’ salivary flow and thus improve appetite. Moreover, a recent review reports that fructose ingestion compared to glucose ingestion is associated with a stronger food-cue reactivity within brain reward regions, and may promote feeding behaviour [83,84,85]. Nevertheless, the dietary fibre in fruits may reduce the metabolic effects on appetite [86]. Fulton et al. have recently reported that protein intake was not influenced by an increase of fruit intake, although consumption moment was not investigated in their study [87]. In comparison with timing of consumption, absence or presence of consumption of protein-rich foods such as dairy products, nuts or seeds appears to be stronger associated with the risk of lower protein intake. Dietary strategies may consider prioritizing and focusing on increasing consumption frequency of protein-rich foods, and then placing the food groups or products at the “right moment”, which appears worthwhile investigating in future research.

### 4.3. Physical Activity Characteristics and Behaviours

Physical activity stimulates skeletal muscle tissue and enables a higher uptake rate of dietary protein in the muscle that increases muscle protein synthesis and muscle mass gain [17]. Regarding the risk of having a lower protein intake, lower levels of physical activity constitute a risk factor for older adults with poor appetite. This group is especially at risk for future functional decline as both protein and physical activity are essential to maintain physical function [88,89]. Vigorous physical activities between lunch and dinner reduce the risk on a lower protein intake in older adults with good appetite. Though it is evidential that physical activity is positively linked to health benefits, it is not realistic for all less active older adults to meet moderate or vigorous physical activity targets [90]. It is recommended to reduce sedentary time and increase light physical activities, and/or schedule physical activity between breakfast and lunch, while placing the protein-rich meal such as a warm meal during daytime, e.g., at lunch [90]. One approach to increase physical activity is related to the psychological aspect, as previous studies found that self-efficacy is consistently linked to physical activity in older adults [91,92], and is a stronger predictor for the actual behaviour compared to other attitudinal measures [49]. Hence, based on our findings related to self-efficacy, strategies should focus on increasing older adults’ confidence in their ability to engage in physical activities even in difficult situations, such as when they are feeling tired, stressed, depressed or anxious, after recovering from an injury or illness that required resting, or after experiencing family problems. Ashford et al. reported that feedback on past or peer-performance, as well as vicarious experience could be effective strategies in increasing self-efficacy towards physical activity [93]. Strategies may also tap into emerging technology or alternative ways to reframe physical activity as fun and healthy rather than work or exercise [94].

Sedentary behaviour (assessed as the number of waking hours of sitting) was not associated with protein intake. However, Uffelen et al. [95] reported that older adults found it difficult to report their sitting duration, this result might thus be biased. Although sleeping duration was also not retained in the final models as a determinant of protein intake, a recent review concluded that the diet of older short sleepers has a lower protein content [96]. In addition, physical activity was positively associated with sleep duration in older adults with depressive symptoms [97]. Thus, the physical activity level can potentially improve sleep [98] and alleviate depressive symptoms [99]; at the same time, improved sleep may also alleviate fatigue [100]. Since older adults with a lower protein intake also experience lower self-efficacy towards physical activity when they feel tired or depressed, strategies that improve sleeping duration and sleep quality may increase the effectiveness of strategies to increase the physical activity level of older adults.

### 4.4. Limitations

To our knowledge, this study is the first attempt of using a large-scale survey to investigate appetite and protein intake of European older adults with a wide range of determinants, covering demographic, socioeconomic, health, knowledge, attitudinal, diet-related and physical activity characteristics and behaviours. Nevertheless, this study faces some limitations that are inherently related to the study population and study methodology. First, the survey results could only be valid if completed by older adults without cognitive impairment [101]. Second, the probability of low protein intake was calculated using the Pro55+. Although the Pro55+ tool has shown satisfactory discriminative properties [44], there could be possible non-differential misclassification, leading to underestimated strength of associations. Third, data were collected using online methods. Thus, our study samples only include older adults with online access and a minimum level of required information-and-communication-technology skills. A recent review reported that older adults recruited by a market research agency for an online survey had a tendency to have a higher education and income level, which may introduce possible selection bias; thus, the results should not be generalized to other populations [102]. Fourth, similar to most consumer research, this study relied on self-reported measures of personal characteristics attitudes, related perceptions, diet-related and physical activity behaviours. Although self-reported and subjective opinions provide valuable insights, they may suffer from social desirability and hypothetical bias, and hence, may deviate from actual behaviour [103]. Finally, although the samples of older populations involved in this study were representative based on gender and region, the study findings primarily apply and should be interpreted taking into account the specific characteristics of the study samples, with potential limitations in terms of generalisation to the overall older population.

## 5. Conclusions

This study provides an overview of the similarities and differences in the profiles of four identified appetite and protein intake strata, habitual dietary and physical activity behaviours that are associated with protein intake, as well as recommendations for developing optimal dietary and physical activity strategies to prevent protein malnutrition in older adults in the EU. To the best of our knowledge, this study represents a pioneer attempt to adopt a holistic approach that covers socioeconomic and demographic background, health characteristics, presence of health problems, knowledge and attitude related to protein, food and diet, attitude towards physical activity, as well as habitual behaviours.

Adequate energy and protein intake and physical activity are commonly regarded as the most promising strategies to prevent protein malnutrition. Our findings suggest that effective dietary strategies to increase protein intake should take into account sensory properties, familiarity, affordability, accessibility and convenience. Older adults’ awareness about the importance of protein consumption should be increased, specifically the amount of protein needed and concrete actions on how recommended intake levels could be achieved (e.g., more frequent consumption of specific protein-rich food products). Consumption of certain foods at a certain moment of the day and physical activity level or pattern were associated with a lower risk of having lower protein intake. Low level of physical activity emerged as a risk factor for having a lower protein intake in older adults with poor appetite, and vigorous physical activities between lunch and dinner were associated with a lower risk in older adults with good appetite. Increasing older adults’ confidence in their ability to engage in physical activities in more difficult situations could be a potentially effective strategy to increase physical activity level, and thereby increase protein intake. Dietary and physical activity strategies to increase protein intake should be tailored according to older adults’ appetite profiles.

## Figures and Tables

**Table 1:** Size and mean scores of the appetite and protein intake strata in 1825 adults aged 65 years and older from five European countries.

Strata	*n* = 1825 (%)	Mean ± SD	
		Appetite Score ^‡^	Probability Protein Intake *
Total sample		15.40 ± 1.91	0.29 ± 0.31
1. appi—Poor appetite and lower level of protein intake	222 (12.2)	12.90 ± 1.51	0.66 ± 0.21
2. APpi—Good appetite but lower level of protein intake	466 (25.5)	16.23 ± 1.13	0.64 ± 0.21
3. apPI—Poor appetite but higher level of protein intake	271 (14.8)	13.13 ± 1.32	0.09 ± 0.08
4. APPI—Good appetite and higher level of protein intake	866 (47.5)	16.30 ± 1.19	0.08 ± 0.08

^‡^ Simplified nutritional appetite questionnaire (SNAQ) scores: poor appetite was defined as having a total SNAQ score below or equal to 14 (total sample mean ± SD = 15.40 ± 1.91); * Probability of protein intake lower than 1.0 g/kg adjusted BW/day; a low level of protein intake was defined as having a probability higher than 0.3 (total sample mean ± SD = 0.30 ± 0.27). SD: Standard deviation.

**Table 2:** Strata profiles based on socio-demographic characteristics in 1825 adults aged 65 years and older from five European countries.

Socio-Demographics (% of Sample)		Total Sample	Strata (%)				*p*-Value (ϕc)
			appi (12.2)	APpi (25.5)	apPI (14.8)	APPI (47.5)	
Gender	Male	50.4	55.4 ^b,c^	60.3 ^c^	45.4 ^a,b^	45.4 ^a^	<0.001 (0.132)
	Female	49.6	44.6 ^a,b^	39.7 ^a^	54.6 ^b,c^	54.6 ^c^	
Age group	Below 70 years	55.9	46.8 ^a^	55.2 ^a,b^	58.7 ^a,b^	57.9 ^b^	0.022 (0.073)
	70 years or above	44.1	53.2 ^b^	44.8 ^a,b^	41.3 ^a,b^	42.1 ^a^	
Country	United Kingdom	20.0	35.6 ^b^	34.4 ^b^	7.4 ^a^	12.2 ^a^	<0.001 (0.224)
	The Netherlands	20.1	13.5 ^a^	26.6 ^b^	15.5 ^a^	19.6 ^a^	
	Spain	19.9	15.3 ^b^	7.7 ^a^	27.3 ^c^	25.5 ^c^	
	Poland	20.0	8.6 ^a^	19.3 ^b,c^	15.5 ^a,b^	24.7 ^c^	
	Finland	20.0	27.0 ^b,c^	12.0^a^	34.3 ^c^	18.0 ^b^	
Education	Below tertiary level	59.6	67.6 ^b^	61.6 ^a,b^	63.8 ^a,b^	55.2 ^a^	0.001 (0.093)
	Tertiary level or above	40.4	32.4 ^a^	38.4 ^a,b^	36.2 ^a,b^	44.8 ^b^	
Main household grocery shopper	Yes	70.3	70.3	66.7	71.6	71.8	0.515 (0.038)
	No	10.1	9.5	12.3	8.5	9.6	
	Shared responsibility	20.3	20.2	21.0	19.9	18.6	
Living alone	Yes	30.6	36.5	30.9	32.5	28.4	0.109 (0.058)
	No ^‡^	69.4	63.5	69.1	67.5	71.6	
Own food decision maker	Yes, always	69.3	69.8 ^a,b^	63.3 ^a^	71.2 ^a,b^	71.8 ^b^	0.029 (0.062)
	Yes, sometimes	25.1	22.5	29.8	23.6	23.7	
	No, someone else decides	5.6	7.7	6.9	5.2	4.5	
Perceived financial situation (*n* = 1791)	Manage quite or very well	45.3	40.6 ^a^	53.1 ^b^	39.1 ^a^	44.2 ^a^	0.001 (0.079)
	Get by alright	38.3	37.0 ^a,b^	33.1 ^a^	43.6 ^b^	39.7 ^a,b^	
	Have some or severe difficulties	15.4	22.4 ^b^	13.8 ^a^	17.3 ^a,b^	16.1 ^a,b^	
Monthly net household income (*n* = 1589)	Below 1000 EUR	20.5	19.6 ^a,b,c^	20.4 ^c^	11.8 ^a^	23.6 ^b,c^	0.001 (0.082)
	Between 1000 EUR and 2000 EUR	37.2	41.8 ^a,b,c^	34.2 ^a^	45.6 ^c^	35.0 ^a,b^	
	2000 EUR or above	42.3	38.6	45.4	42.6	41.4	

Strata are listed in abbreviated form, appi: poor appetite and lower level of protein intake; APpi: good appetite but lower level of protein intake; apPI: poor appetite but higher level of protein intake; and APPI: good appetite and higher level of protein intake. The superscripts ^a–c^ indicate significantly different levels or proportions across the four strata (across rows) at the 0.05 level in ascending order. Cramer’s phi (ϕc) indicates the multitude of effect size. ^‡^ The strata did not differ significantly in terms of the number of household member(s); the difference was only between single or non-single household.

**Table 3:** Strata profiles based on health characteristics in 1825 adults aged 65 years and older from five European countries.

Health Characteristics (% of Sample)		Total Sample	Strata (%)				*p*-Value (ϕc)
			appi (12.2)	APpi (25.5)	apPI (14.8)	APPI (47.5)	
MUST—risk levels of malnutrition (*n* = 1659)	Low	74.8	69.4 ^a,b^	80.7 ^c^	60.8 ^a^	77.4 ^b,c^	<0.001 (0.131)
	Medium	18.7	19.4 ^a,b^	15.3 ^a^	25.2 ^b^	18.3 ^a,b^	
	High	6.5	11.2 ^b^	4.0 ^a^	14.0 ^b^	4.3 ^a^	
Ability to walk or move own wheelchair for more than 5 minutes (*n* = 1822) ^#^	Yes without difficulties	87.0	74.2 ^a^	89.0 ^b,c^	82.3 ^a,b^	90.8 ^c^	<0.001 (0.125)
	Yes with difficulties	9.3	19.5 ^b^	6.5 ^a^	11.8 ^a,b^	7.4 ^a^	
	No or only able with help	3.7	6.3 ^b^	4.5 ^b^	5.9 ^b^	1.8 ^a^	
Ability to prepare own warm meal (*n* = 1781) ^‡^	Yes without difficulties	89.5	85.5 ^a^	92.5 ^b^	89.0 ^a,b^	93.8 ^b^	<0.001 (0.084)
	Yes with difficulties	4.9	7.0	4.0	7.2	4.4	
	No or only able with help	5.6	7.5 ^b^	3.5 ^a,b^	3.8 ^a,b^	1.8 ^a^	

Strata are listed in abbreviated form, appi: poor appetite and lower level of protein intake; APpi: good appetite but lower level of protein intake; apPI: poor appetite but higher level of protein intake; and APPI: good appetite and higher level of protein intake. The superscripts ^a–c^ indicate significantly different levels or proportions across the four strata (across rows) at the 0.05 level in ascending order. Cramer’s phi (ϕc) indicates the multitude of effect size. ^#^ Users of electrical wheelchair or participants who never walk outside for 5 min were excluded (*n* = 3). ^‡^ The proportion of female able to prepare their own meal without difficulties was significantly larger than male. MUST: Malnutrition Universal Screening Tool.

**Table 4:** Strata profiles based on health problems in 1825 adults aged 65 years and older from five European countries.

Presence of Health Problem(s) (% of Sample)	Total Sample	Strata (%)				*p*-Value (ϕc)
		appi (12.2)	APpi (25.5)	apPI (14.8)	APPI (47.5)	
Pain in mouth, teeth or gums (*n* = 1808)	8.5	8.7 ^a,b,c^	6.9 ^a^	13.3 ^c^	7.7 ^a,b^	0.016 (0.076)
Dry mouth (*n* = 1817)	16.3	21.4 ^b^	11.2 ^a^	21.8 ^b^	16.0 ^a,b^	<0.001 (0.102)
Difficulty swallowing (*n* = 1821)	3.2	6.8 ^b^	1.9 ^a^	5.2 ^a,b^	2.3 ^a^	0.001 (0.097)
Difficulty chewing (*n* = 1821)	5.3	8.6 ^b,c^	4.3 ^a,b^	9.2 ^c^	3.8 ^a^	0.001 (0.099)
Cardiovascular disease (*n* = 1812)	24.1	23.6	23.1	28.1	23.5	0.434 (0.039)
Hypertension (*n* = 1815)	42.4	42.7	39.0	43.7	43.8	0.372 (0.042)
Irritable bowel syndrome (*n* = 1816)	12.3	16.7 ^b^	8.4 ^a^	14.8 ^a,b^	12.5 ^a,b^	0.007 (0.082)
Other digestive problems (*n* = 1815)	10.2	14.9 ^b^	8.2 ^a^	12.7 ^a,b^	9.4 ^a,b^	0.020 (0.074)
Diabetes/High blood sugar levels (*n* = 1820)	19.2	23.6 ^a,b,c^	17.0 ^a^	26.6 ^c^	17.0 ^a,b^	0.001 (0.095)
High blood cholesterol (*n* = 1813)	29.5	33.5	27.9	29.6	29.2	0.514 (0.036)
Cancer (*n* = 1812)	5.5	6.8 ^a,b^	3.7 ^a^	8.5 ^b^	5.1 ^a,b^	0.033 (0.069)
Food allergy (*n* = 1820)	6.7	7.7	6.9	8.9	5.7	0.278 (0.046)
Food intolerance (*n* = 1819)	7.2	9.5	6.0	10.0	6.4	0.083 (0.061)
Chronic kidney disease (*n* = 1820)	2.3	4.1	1.9	3.7	1.5	0.041 (0.067)
Other chronic diseases or pain in general (*n* = 1811)	20.5	23.3 ^a,b,c^	19.2 ^b^	28.1 ^c^	18.2 ^a,b^	0.003 (0.088)

Strata are listed in abbreviated form, appi: poor appetite and lower level of protein intake; APpi: good appetite but lower level of protein intake; apPI: poor appetite but higher level of protein intake; and APPI: good appetite and higher level of protein intake. The superscripts ^a–c^ indicate significantly different levels or proportions across the four strata (across rows) at the 0.05 level in ascending order. Cramer’s phi (ϕc) indicates the multitude of effect size.

**Table 5:** Strata profiles based on knowledge of and attitude towards protein, food and diet in 1825 adults aged 65 years and older from five European countries.

Knowledge and Attitude (% of Sample)		Total Sample	Strata (%)				*p*-Value (ϕc)
			appi (12.2)	APpi (25.5)	apPI (14.8)	APPI (47.5)	
Claiming to know what dietary protein is	Yes	64.7	53.2 ^a^	59.9 ^a,b^	66.1 ^b,c^	69.7 ^c^	<0.001 (0.123)
	No	35.3	46.8 ^c^	40.1 ^b,c^	33.9 ^a,b^	30.3 ^a^	
Objective knowledge score about dietary protein (medians) (effect size: η_p_^2^) (*n* = 1180)		5.00	3.00 ^a^	4.00 ^a^	5.00 ^b^	5.00 ^b^	<0.001 (0.021)
Food fussiness (medians) (effect size: η_p_^2^)		2.29	2.57 ^b^	2.29 ^a^	2.43 ^b^	2.29 ^a^	<0.001 (0.026)
Perceived amount of protein in current diet	Too much	7.0	3.2	6.7	6.6	8.2	<0.001 (0.111)
	Just about right	74.4	58.1 ^a^	64.6 ^a^	61.3 ^a^	73.3 ^b^	
	Too little	15.1	24.7 ^b^	13.9 ^a^	22.1 ^b^	11.0 ^a^	
	Do not know	10.5	14.0 ^b^	14.8 ^b^	10.0 ^a,b^	7.5 ^a^	
Intention to change the amount of protein in diet	Yes, increase	10.0	10.8 ^a,b^	7.7 ^a^	14.0 ^b^	9.7 ^a,b^	<0.001 (0.081)
	Yes, decrease	4.9	2.7	4.7	4.4	5.8	
	No, remain the same	66.4	59.5 ^a^	67.0 ^a,b^	59.0 ^a^	70.1 ^b^	
	Do not know	18.7	27.0 ^b^	20.6 ^b^	22.5 ^b^	14.4 ^a^	
Would increase amount of protein in diet if told by (% yes)	Health professional	76.2	67.6 ^a^	77.9 ^b^	73.1 ^a,b^	78.4 ^b^	0.002 (0.075)
	Food industry	4.8	3.6	2.8	6.6	5.7	0.086 (0.055)
	Family	21.7	23.0 ^a,b^	17.4 ^a^	27.3 ^b^	21.9 ^a,b^	0.041 (0.060)
	Friends	15.7	15.8 ^a,b^	11.8 ^a^	19.2 ^b^	16.6 ^a,b^	0.051 (0.059)

Strata are listed in abbreviated form, appi: poor appetite and lower level of protein intake; APpi: good appetite but lower level of protein intake; apPI: poor appetite but higher level of protein intake; and APPI: good appetite and higher level of protein intake. The superscripts ^a–c^ indicate significantly different levels or proportions across the four strata (across rows) at the 0.05 level in ascending order. Cramer’s phi (ϕc) or partial eta-squared (η_p_^2^) indicate the multitude of effect size.

**Table 6:** Strata profiles based on attitude towards physical activity in 1825 adults aged 65 years and older from five European countries.

Physical Activities (% of Sample)		Total Sample	Strata (%)				*p*-Value (ϕc)
			appi (12.2)	APpi (25.5)	apPI (14.8)	APPI (47.5)	
Willingness to change daily physical activity pattern	Yes	32.1	33.3	31.1	32.1	32.3	<0.001 (0.112)
	No	34.0	23.0 ^a^	27.3 ^a^	38.7 ^b^	39.0 ^b^	
	Not applicable	33.9	43.7 ^b^	41.6 ^b^	29.2 ^a^	28.6 ^a^	
Self-efficacy towards physical activity (medians) (effect size: η_p_^2^)		2.87	2.47 ^a^	3.00 ^b^	2.73 ^a^	2.93 ^b^	<0.001 (0.035)

Strata are listed in abbreviated form, appi: poor appetite and lower level of protein intake; APpi: good appetite but lower level of protein intake; apPI: poor appetite but higher level of protein intake; and APPI: good appetite and higher level of protein intake. The superscripts ^a–b^ indicate significantly different levels or proportions across the four strata (across rows) at the 0.05 level in ascending order. Cramer’s phi (ϕc) or partial eta-squared (η_p_^2^) indicate the multitude of effect size.

**Table 7:** Association between behavioural determinants and probability of having a protein intake lower than 1.0 g/kg adjusted BW/day stratified by appetite level in 1825 adults aged 65 years and older from five European countries.

	Poor Appetite (Model 1, *n* = 485)					Good Appetite (Model 2, *n* = 1263)				
	b	SE	β	BCa 95% CI		b	SE	β	BCa 95% CI	
				Lower	Upper				Lower	Upper
(Constant)	0.155 **	0.027		0.103	0.208	0.252 **	0.017		0.218	0.291
Food expenses less than 60 EUR per week^#^	0.104 *	0.029	0.157	0.050	0.162	0.041 *	0.017	0.070	0.009	0.076
Smoking						−0.061 *	0.019	−0.081	−0.100	−0.024
**Diet-related habits**										
No consumption of milk or yogurt	0.193 **	0.039	0.231	0.112	0.272	0.163 **	0.025	0.191	0.117	0.213
No consumption of nuts or seeds	0.124 **	0.028	0.186	0.070	0.180	0.171 **	0.017	0.280	0.138	0.205
Consumption of warm meal during lunch						−0.126 **	0.015	−0.222	−0.155	−0.097
Consumption of soup during mid-afternoon snack						−0.078 *	0.030	−0.068	−0.134	−0.015
Consumption of milk or yogurt during mid-afternoon snack						−0.060 *	0.019	−0.072	−0.098	−0.023
Consumption of fruits during dinner	−0.117 **	0.034	−0.144	−0.183	−0.055					
Consumption of dessert during dinner	0.084 **	0.027	0.127	0.033	0.135					
Consumption of cold meal during evening snack	−0.094 *	0.031	−0.121	−0.152	−0.034	−0.073 **	0.018	−0.092	−0.109	−0.040
Consumption of fruits during evening snack						0.045 *	0.017	0.074	0.012	0.078
**Physical activity**										
Low physical activity level	0.180 **	0.037	0.229	0.104	0.252					
Moderate physical activity level	0.073 *	0.029	0.108	0.022	0.133					
Performing vigorous physical activities between lunch and dinner						−0.054 *	0.018	−0.072	−0.088	−0.017

** *p* ≤ 0.001; * *p* < 0.05 based on robust method with 1000 bootstrap samples; Model goodness-of-fit: *R*^2^ adjusted = 20.6% (model 1); 21.9% (model 2); b: unstandardized coefficient estimate; SE: standard error; β: standardized coefficient estimate; BCa 95% CI: bootstrapped 95% confidence-interval based on bias-corrected and accelerated method. Baseline categories: High (physical activity level); Between 60 and 119 EUR (food expenses); dummy variable entered but not shown in the models; Food expenses, 120 EUR or above per week (model 1: b = 0.043, *p*-value = 0.414; model 2: b = −0.018, *p*-value = 0.447); Food expenses, prefer not to say or do not know (model 1: b = 0.024, *p*-value = 0.550; model 2: b = 0.019, *p*-value = 0.384). ^#^ calculated based on the sum of expenditures on foods consumed at home (bought from the supermarkets or shops) and foods consumed out of home (meals, snacks or drinks from restaurants or canteens, etc.). Excluded variables (selection based on backward and forward stepwise regressions): Hours of sleeping per day (continuous), Hours of sitting per day (continuous), Diet status (dichotomous), Alcohol use (dichotomous), Consumption pattern of foods (two out of nine items were not retained in the models: cereals like cornflakes or muesli, biscuits or cookies) (dichotomous in eight options: breakfast, mid-morning snack, lunch, mid-afternoon snack, dinner, evening snack, nocturnal eating and I do not consume this food), physical activity patterns (two out of three items were not retained in the models: walking, moderate PA) (dichotomous in four options: before breakfast, between breakfast and lunch, between lunch and dinner and after dinner).

## Appendix A

**Table A1:** Consumption pattern of 10 food items (*n* = 1825).

Food Items (% yes)	Breakfast	Mid-Morning Snack	Lunch	Mid-Afternoon Snack	Dinner	Evening Snack	Nocturnal Eating	I Do Not Consume This Food
Cereals like cornflakes or muesli	40.9	3.6	1.5	2.7	2.6	4.1	0.7	48.4
Dairy or plant-based milk or yogurt	40.4	9.3	18.6	13.9	21.6	14.7	2.6	13.9
Soup	0.9	0.5	56.5	7.4	33.0	1.6	1.3	8.9
Warm meal	5.1	0.5	48.5	3.9	64.5	1.3	1.5	0.5
Cold meal	19.2	10.1	38.0	11.7	29.1	17.7	2.4	5.6
Dessert	0.5	3.3	25.1	14.2	41.6	8.2	1.1	22.8
Biscuits or cookies	8.8	16.8	3.7	30.3	2.6	24.3	2.0	29.3
Fruits	15.7	25.9	25.4	43.4	20.5	31.7	2.7	3.3
Nuts or seeds	6.4	10.1	4.7	24.4	4.4	30.1	1.8	32.5

The sum of percentage in response is not equal to 100% per food item, because participants could choose more than one moment of the day for each food item.

**Table A2:** Strata profiles based on the misconception about dietary protein (*n* = 1180).

Misconception about Dietary Protein—Proportion of Participants Who Indicated True or False to the Statements Incorrectly (or Did not Know the Answer) (*n* = 1180) (% of Sample)	Total Sample	Strata (%)				
		appi (12.2)	APpi (25.5)	apPI (14.8)	APPI (47.5)	*p*-Value (ϕc)
“You need protein in the diet for energy”	18.0	16.9	20.1	16.8	17.5	0.760 (0.031)
“You need protein in the diet for repairing bones and muscles”	10.7	9.3	11.8	10.6	10.4	0.884 (0.024)
“You need protein in the diet for building body cells”	12.9	13.6	12.9	14.5	12.3	0.875 (0.024)
“Health experts recommend people of my age to consume less protein” *	48.7	50.0	53.8	42.5	48.0	0.119 (0.070)
“The human body is good at storing protein to use later, it is thus not necessary to consume a steady amount of protein every day” *	47.4	54.2	49.5	41.9	46.7	0.173 (0.065)
“One meal per day with a good protein source is sufficient” *	74.6	83.1 ^b^	78.9 ^a,b^	76.0 ^a,b^	70.5 ^a^	0.006 (0.103)
“Cooked lean beef has more protein than the same amount of cooked tomato”	37.1	38.1	39.4	35.8	36.3	0.796 (0.029)
“Whole milk (100 mL) has more protein than cheese (100 g)” *	44.7	46.6 ^a,b^	51.6 ^b^	43.6 ^a,b^	41.6 ^a^	0.045 (0.083)
“You will experience loss in muscle mass if you do not consume enough protein”	27.5	28.8	33.0	25.1	25.3	0.101 (0.073)

Strata are listed in short form, appi: poor appetite and lower level of protein intake; APpi: good appetite but lower level of protein intake; apPI: poor appetite but higher level of protein intake; and APPI: good appetite and higher level of protein intake. The superscripts ^a–b^ indicate significantly different levels or proportions across the four strata (across rows) at the 0.05 level in ascending order. Cramer’s phi (ϕc) indicates the multitude of effect size. * False statements (i.e., “No” is the correct answer to these statements).
